# Generalized erythematous papules in an adult male

**DOI:** 10.1016/j.jdcr.2023.11.035

**Published:** 2023-12-30

**Authors:** Ma. Isabela P. Ong, Patricia Anne T. Tinio, Jose Giovanni E. Dimayuga, Reagan Grey T. Reyes, Maria Jasmin J. Jamora, Maria Bettina Teresa G. Pascual

**Affiliations:** a Resident, Department of Dermatology, Makati Medical Center, Makati City, Metro Manila, Philippines; b Active Consultant, Department of Dermatology, Makati Medical Center, Makati City, Metro Manila, Philippines

**Keywords:** cutaneous manifestations of HIV, disseminated histoplasmosis, mucocutaneous infections

## Case report

**Fig 1:**
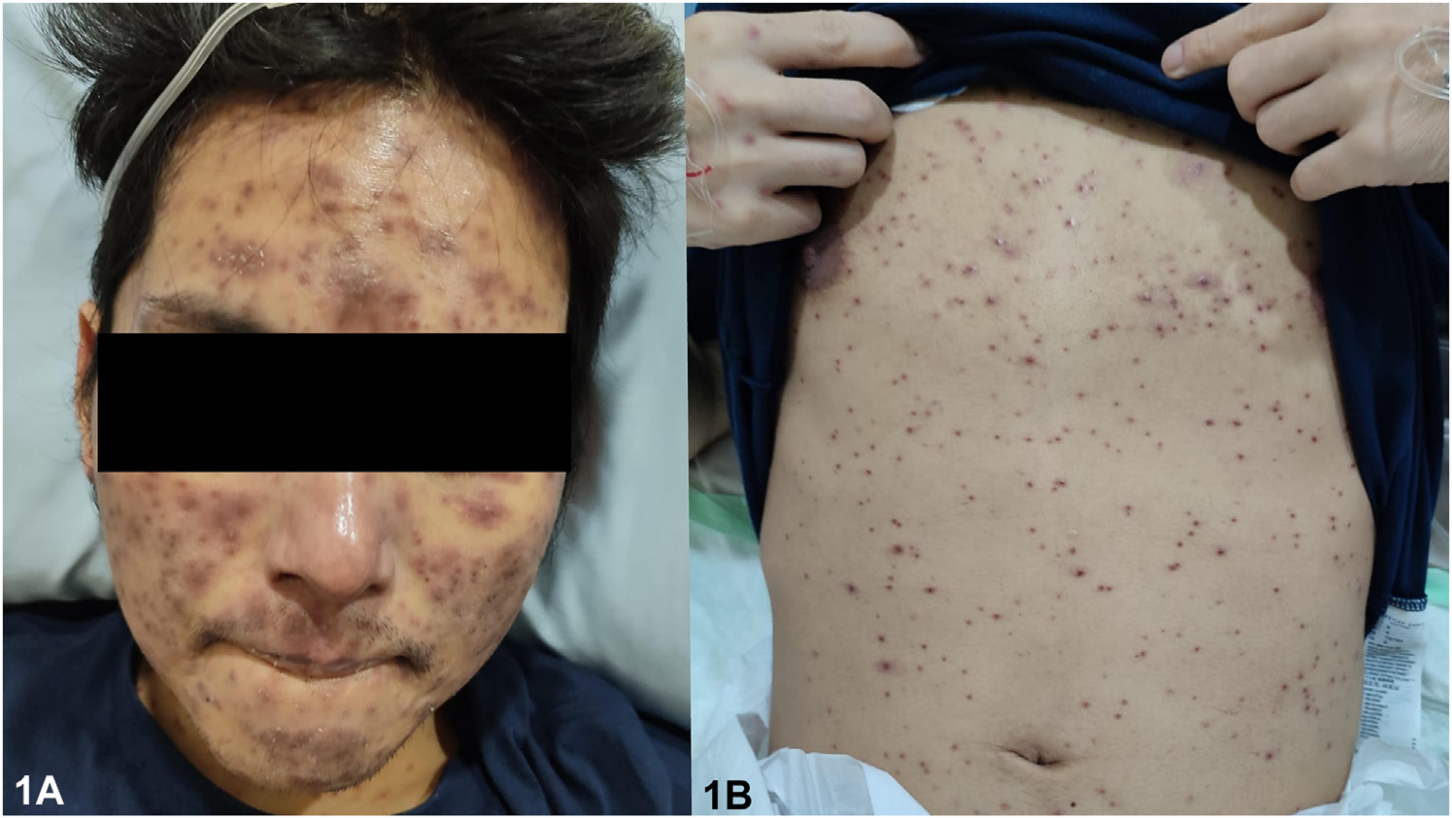

**Fig 2:**
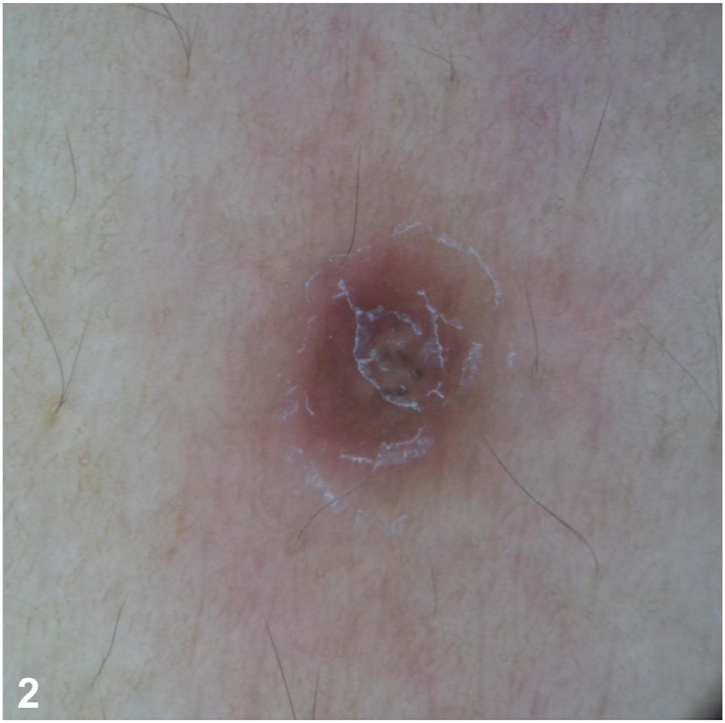

**Fig 3:**
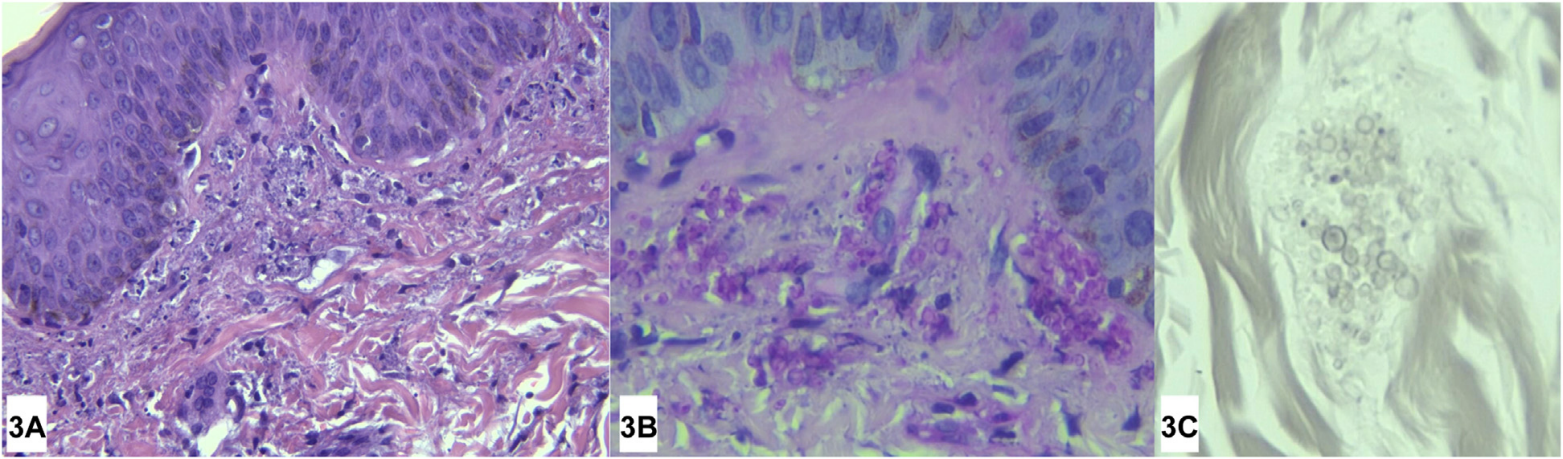

This is a case of a 31-year-old Filipino male managed as an immunocompromised host (HIV) with a CD4 count of 73 cells/mm^3^ already being managed for a 2 month history of gastrointestinal tuberculosis. Despite treatment with Anti-Koch’s medication, he also developed cough, decreased breath sounds, and febrile episodes. This was associated with generalized erythematous papules (Fig 1) which, on dermoscopy, showed white scales, brown globules, and pink areas (Fig 2). Upon further work-up, fungal elements were seen on peripheral blood smear, and skin punch biopsy showed systemic fungal infection staining positively for fungal elements on Periodic Acid Schiff and Gomori Methenamine 30 Silver stains (Fig 3).

**Question 1: Which would be your most likely diagnosis?**

A. Sporotrichosis
B. Histoplasmosis
C. Chromoblastomycosis
D. Coccidioidomycosis
E. Phaeohyphomycosis

**Answers:**

A. Sporotrichosis – Incorrect. Fungal elements for Sporotrichosis would also stain positively on PAS and GMS staining with polymorphonuclear infiltrates, however it would show oval/cigar shaped yeasts.^1^
B. Histoplasmosis – Correct. Histopathology for cases with HIV will show a lack of granulomatous response and instead show mild perivascular and interstitial infiltrate with polymorphonuclear cells, and presence of fungal spores that will stain positively with both PAS and GMS. However, in some cases biopsies from extremely immunosuppressed patients may have a minimal immune response.^2^
C. Chromoblastomycosis – Incorrect. Chromoblastomycosis would also stain positively for PAS and GMS staining. However, this would show epidermal acanthosis with neutrophil microabscesses. The dermis shows abscess formation, necrosis, granulomatous, and mixed inflammatory infiltrate.^2^
D. Coccidioidomycosis – Incorrect. Coccidioidomycosis shows suppuration and pseudoepitheliomatous hyperplasia with dermal perivascular infiltrates on histopathology.^2^
E. Phaeohyphomycosis – Incorrect. Phaeohyphomycosis presents with septated branched or unbranched hyphae. These can be found in necrotic centers surrounded by macrophages, giant cells, and neutrophils. PAS and GMS staining will highlight the fungal elements upon further staining.^2^

**Question 2: Which of the statements regarding the pathogenesis of Histoplasmosis is correct?**

A. The spread of *Histoplasma capsulatum* is solely via the lymphatic system
B. Patients with a CD4 count of >500 cells/uL are susceptible to invasive fungal infections
C. Person-to-person transmission of Histoplasmosis is common
D. *Histoplasma* can cause infections in both animals and humans
E. *Histoplasma capsulatum* maintains its yeast form from inhalation to dissemination

**Answers:**

A. The spread of *Histoplasma capsulatum* is solely via the lymphatic system – Incorrect. *Histoplasma capsulatum* is phagocytosed and macrophages spread the infection initially through the lymphatic system, then via hematogenous spread through reticuloendothelial system.^3^
B. Patients with a CD4 count of >500 cells/uL are susceptible to invasive fungal infections – Incorrect. Patients with a CD4 count of >500 cells/uL are susceptible to Acute retroviral syndrome, Herpes zoster infection, and Seborrheic Dermatitis. Invasive fungal infections are common in patients with CD4 count of less than 200 cells/uL.^1^
C. Person-to-person transmission of Histoplasmosis is common – Incorrect. Person-to-person infection happens only rarely due to organ transplantation. It is primarily acquired through the environment.^3^
D. *Histoplasma* can cause infections in both animals and humans – Correct. Disseminated Histoplasmosis infection of horses is caused by *Histoplasma farciminosum,* while human infections are caused by either a classic or small-form histoplasmosis and African histoplasmosis brought about by 2 variants: *H. capsulatum* var. *capsulatum* and *H. capsulatum* var. *duboisii.*^1^
E. *Histoplasma capsulatum* maintains its yeast form from inhalation to dissemination – Incorrect. *Histoplasma capsulatum* is dimorphic fungus inhaled as microconidia or hyphal fragment. It travels through the respiratory system, and in the alveoli, turns into yeast form.^3^

**Question 3: Which of the following is true in the treatment of Histoplasmosis?**

A. Itraconazole is indicated for 1 week of mild acute pulmonary histoplasmosis
B. Liposomal amphotericin B is given to patients with moderately severe to severe acute pulmonary disease
C. Fluconazole is more effective for Histoplasmosis than Itraconazole
D. Fluconazole is the recommended treatment for patients who are pregnant
E. The dose for Amphotericin B given to patients with widespread and severe infections is 10 mg/kg/day

**Answers:**

A. Itraconazole is indicated for 1 week of mild acute pulmonary histoplasmosis – Incorrect. There is no indication for treatment if the disease is classified as mild acute pulmonary histoplasmosis, and if the symptoms surpass 4 weeks or there is history of immunocompromisation, patients are treated with Itraconazole.^3^
B. Liposomal amphotericin B is given to patients with moderately severe to severe acute pulmonary disease – Correct. For moderately severe to severe acute pulmonary disease, treatment is liposomal amphotericin B, which is shifted to itraconazole once improved.^3^
C. Fluconazole is more effective for Histoplasmosis than Itraconazole – Incorrect. Fluconazole is less effective for histoplasmosis, It is only an alternative for those intolerant of itraconazole, with moderate effectivity.^4^
D. Fluconazole is the recommended treatment for patients who are pregnant – Incorrect. Amphotericin B is preferred and azole antifungals should be avoided, especially in the first trimester as some case reports have shown that they may cause congenital malformations.^5^
E. The dose for Amphotericin B given to patients with widespread and severe infections is 10 mg/kg/day – Incorrect. The dose for Amphotericin B given to patients with widespread and severe infections is 1 mg/kg/day. Alternatives include Posaconazole and Voriconazole.^1^

## Conflicts of interest

None disclosed.
